# The Efficacy of Silver-Based Electrospun Antimicrobial Dressing in Accelerating the Regeneration of Partial Thickness Burn Wounds Using a Porcine Model

**DOI:** 10.3390/polym13183116

**Published:** 2021-09-15

**Authors:** Thien Bui-Thuan Do, Tien Ngoc-Thuy Nguyen, Minh Hieu Ho, Nghi Thi-Phuong Nguyen, Thai Minh Do, Dai Tan Vo, Ha Thi-Ngoc Hua, Thang Bach Phan, Phong A. Tran, Hoai Thi-Thu Nguyen, Toi Van Vo, Thi-Hiep Nguyen

**Affiliations:** 1 Department of Tissue Engineering and Regenerative Medicine, School of Biomedical Engineering, International University, Ho Chi Minh City 700000, Vietnam; moonlightshadow1810@gmail.com (T.B.-T.D.); nntt0001@gmail.com (T.N.-T.N.); hohieuminh96@gmail.com (M.H.H.); nghinguyen.295@gmail.com (N.T.-P.N.); thaiminhdo@gmail.com (T.M.D.); vvtoi@hcmiu.edu.vn (T.V.V.); 2 Vietnam National University, Ho Chi Minh City 700000, Vietnam; pbthang@inomar.edu.vn (T.B.P.); ntthoai@hcmiu.edu.vn (H.T.-T.N.); 3 Veterinary Hospital, Faculty of Animal Sciences and Veterinary Medicine, Nong Lam University, Ho Chi Minh City 70000, Vietnam; dai.votan@hcmuaf.edu.vn; 4 Department of Anatomic Pathology, University of Medicine and Pharmacy, Ho Chi Minh City 700000, Vietnam; hthngocha@umc.edu.vn; 5 Center for Innovative Materials and Architectures (INOMAR), Vietnam National University, Ho Chi Minh City 700000, Vietnam; 6 Centre for Biomedical Technologies, Queensland University of Technology (QUT), 60 Musk Avenue, Kelvin Grove, Brisbane, QLD 4059, Australia; phong.tran@qut.edu.au; 7 Interface Science and Materials Engineering Group, School of Mechanical, Medical and Process Engineering, QUT, 60 Musk Avenue, Kelvin Grove, Brisbane, QLD 4059, Australia; 8 School of Biotechnology, International University, Ho Chi Minh City 700000, Vietnam

**Keywords:** wound dressing, electrospinning, polycaprolactone, gelatin, silver nanoparticle, antibacterial property, burn, pig model

## Abstract

(1) Background: Wounds with damages to the subcutaneous are difficult to regenerate because of the tissue damages and complications such as bacterial infection. (2) Methods: In this study, we created burn wounds on pigs and investigated the efficacy of three biomaterials: polycaprolactone-gelatin-silver membrane (PCLGelAg) and two commercial burn dressings, Aquacel^®^ Ag and UrgoTul^TM^ silver sulfadiazine. In vitro long-term antibacterial property and in vivo wound healing performance were investigated. Agar diffusion assays were employed to evaluate bacterial inhibition at different time intervals. Minimum inhibitory concentration (MIC), minimum bactericidal concentration (MBC) and time-kill assays were used to compare antibacterial strength among samples. Second-degree burn wounds in the pig model were designed to evaluate the efficiency of all dressings in supporting the wound healing process. (3) Results: The results showed that PCLGelAg membrane was the most effective in killing both Gram-positive and Gram-negative bacteria bacteria with the lowest MBC value. All three dressings (PCLGelAg, Aquacel, and UrgoTul) exhibited bactericidal effect during the first 24 h, supported wound healing as well as prevented infection and inflammation. (4) Conclusions: The results suggest that the PCLGelAg membrane is a practical solution for the treatment of severe burn injury and other infection-related skin complications.

## 1. Introduction

Burn injuries are mostly caused by unintentional accidents globally and commonly happen in developing countries where primary care is practically insufficient [1]. Burn trauma represents the type of injury that can be caused by heat, freezing, electricity, chemicals, and radiation. Among all types of burn causes, thermal burn, which results from direct contact with an intense heat source, is the most common [2,3]. When tissues are exposed to excessively high temperatures, protein denaturing and cellular destruction are induced, resulting in local or systemic effects [4,5,6,7]. Patients with serious thermal burn injury are usually associated with a high rate of morbidity or even mortality if they do not receive appropriate treatment in time [6,7]. Burn injuries are classified based on the depth and level of the damaged skin layer. When burning occurs at the first layer of skin, the epidermal layer, it generally takes about 3 to 7 days to completely heal without inflammation and infection. However, when the damage reaches the dermal layer, including superficial partial- and deep partial-thickness (second-degree burn), it takes at least 3 weeks to heal, with a high risk of infection and scar formation. In third-degree burns, which involve damage to the subcutaneous fat or deeper of full skin thickness, skin cannot regenerate by itself and hence skin graft is required [4,7,8,9].

From the second degree, a burn wound is considered severe and needs specialized care to prevent future complications [9,10]. One of the most challenging problems of burn wound care is infection which happens when the body’s natural defense against external pathogens nearly loses its function [2,3,6,11]. During the wound healing process, exudate production is needed to keep the wound moisturized and transport factors that promote wound healing. However, excessive body fluid secreted on the burnt surface is a favorable environment for bacteria to colonize and proliferate. Therefore, managing wound exudate is the top priority in preventing wound infection [3,6,11], and dressings with moderate or high absorptive capacity of body fluid are commonly employed to keep the wound bed dry and free from bacteria. This practice usually requires frequent changing of dressings depending on the amount of excreted body fluid. However, even in the most aseptic environment, deficient covering of dressing on a deep irregularly-shaped burn wound bed and frequent dressing changes may still induce risks of infection to the vulnerable damaged area. Therefore, the use of antimicrobial agents is extensively combined in dressings as an aid to prevent infection effectively in burn wound treatment. These agents could be antibiotics (tetracycline, gentamicin and methicillin), natural products (honey, essential oil, chitosan), or antiseptic agents [12,13,14].

One of the most prominent antimicrobial agents often incorporated in wound dressings is silver. Although its usefulness for wound treatment has been known since 69 B.C., silver ions have gained recent popularity in infection treatment due to their favorable broad-spectrum coverage, especially in antibiotic-resistant organisms [15]. A number of silver compounds were developed to capitalize on its wound healing benefits. Particularly, in 1967, silver sulfadiazine (SSD), a complex silver salt substance, was first applied as a topical antibiotic to prevent infection in second- and third-degree burns [16]. Later, tentative evidence has found other antibiotics to be more effective; therefore, SSD is no longer generally recommended. Still, for more than 40 years, SSD has remained the gold standard for burn treatment [17]. Some commercial dressing products today still contain SSD such as UrgoTul, NeuSkin-FS and Silvadene. Since the 1990s, when antibiotic resistance becoming more and more threatening to global health, the bactericidal application of silver has been reused and strongly exploited [18]. Currently, there are many commercial products containing silver that are widely used in clinical practice for treating burn injuries. These products vary in form, size, and shape of silver to the derivatives that are incorporated in dressings which contain nanocrystalline silver with sustained release of silver ions into the wound (e.g., Acticoat^®^, Contreet ^®^ Foam), advanced hydrofiber dressings embedded silver ion (e.g., Aquacel Ag^®^) or dressings with silver bound to activated charcoal (e.g., Actisorb Silver^®^). The various silver-containing dressings act by absorbing exudates and releasing silver into the wound bed. Among several forms of silver, silver nanoparticles (AgNPs) have currently gained significant interest as they possess outstanding effectiveness and improved safety compared to other types. In reports from recent years, important conclusions were made that nanocrystalline silver is most useful in infected wounds while SSD may induce slower epithelialization, more infections and more pain in burns [19,20]. In another study, A. Galandakova et al. found that the released Ag^+^ ions are more toxic than AgNPs on human fibroblasts and epidermal keratinocytes in vitro [21]. Besides, AgNPs are effective at killing and preventing the growth of bacteria of both positive and negative Grams, such as *E. coli* (diarrhea), *S. aureus* (skin infection) and *P. aeruginosa* (otitis media), which shows their promising usefulness in various applications [22]. Significantly, AgNPs have also been shown to have a potential in treating full-thickness skin burn in mice [23]. However, many controversies have emerged around the use of silver and AgNPs in medical applications, especially in burn treatment, and that misusing AgNPs may cause adverse effects. In particular, a recent study on the toxicity of silver nanoparticles to wound healing indicated that AgNPs coated in wound dressings impaired tissue regeneration at the phases of epithelialization and blastema formation at the initial healing stage [24]. Another research conducted by L. Cuttle et al. reported that silver dressing delayed or reduced skin re-growth after burn injury [25]. Therefore, a wound dressing containing silver must be investigated thoroughly in research to be proven safe and effective before being applied in clinical practice.

In previous studies, we have fabricated an innovative electrospun polycaprolactone (PCL) membrane coated with multi-layers of gelatin (Gel) carrying AgNPs by immersing technique [26,27,28]. The PCLGelAg membrane (at third coating time) was proven to have sufficient tensile strength, excellent absorptive capacity, in vitro biocompatibility and effective bactericidal property on both Gram-positive and Gram-negative bacteria. Therefore, in this follow-up study, we continued to investigate the long-term in vitro antibacterial efficiency of the selected PCLGelAg membrane and evaluate its in vivo performance to treat partial-thickness burns on a porcine model. Two FDA-approved commercial wound dressing products containing silver, Aquacel Ag^®^ (Aquacel) (ConvaTec, Deeside, UK) and UrgoTul^®^ SSD (UrgoTul) (Urgo Medical, Paris, France), were used as standard references. 

## 2. Materials and Methods

### 2.1. Material

Gelatin type A (extracted from porcine skin, 300 g Bloom) and poly(ε-caprolactone) (PCL) (M_n_ = 80,000) were purchased from Sigma-Aldrich (St Louis, MO, USA). Silver nitrate (AgNO_3_ ≥ 99%) and Acetone (AC, ≥99.5%) solutions were obtained from Xilong Chemical Co., Ltd. (Shantou, China). Mueller-Hinton (MH) was supplied by Hi-Media (Maharastra, India). Two strains of pathogens, *Pseudomonas aeruginosa* ATCC 9027 (*P. aeruginosa*) and *Staphylococcus aureus* ATCC 25,923 (*S. aureus*), were obtained from Marine Laboratory, International University-HCM Vietnam National University, Ho Chi Minh City, Vietnam. The two commercial dressings chosen for this experiment are Aquacel^®^ Ag (ConvaTec, Deeside, UK) (Aquacel) and UrgoTul^®^ SSD (Urgo Medical, Paris, France) (UrgoTul). Histological staining chemicals including Hematoxylin and Eosin (H&E) and Masson’s Trichrome (MT) kits were also purchased from Sigma-Aldrich. 

### 2.2. Antibacterial Activity

#### 2.2.1. Agar Diffusion 

The agar diffusion test was designed with small modifications to show the antibacterial activity of PCLGelAg, Aquacel, and UrgoTul dressings against two bacteria strains: *P. aeruginosa* ATCC 9027 (Gram-negative) and *S. aureus* ATCC 25,923 (Gram-positive). Sample dressings were cut into round disks (0.8 mm in diameter) using an aseptic bio punch and sterilized under UV light for 20 min. The inoculum of each bacteria strain was incubated for 24 h at 37 °C and was diluted to achieve turbidity equivalent to a 0.5 McFarland standard (1 × 10^8^–2 × 10^8^ CFU/mL). Next, 100 μL diluted suspensions were added and spread evenly on Mueller–Hinton (MH) agar surface. After the disks were set, the plate was incubated at 37 °C. The plates were divided into two groups. In the first group, samples were transferred to a new bacteria-rich plate every 24 h for 3 days. In the second group, the plates were left untouched for 7 days at 37 °C. The inhibition zones of both groups (including the diameter of the disk) were determined after each 24 h of incubation and measured using a ruler.

#### 2.2.2. Broth Microdilution Method

The minimum inhibitory concentration (MIC) and minimum bactericidal concentration (MBC) of the aforementioned three dressings were determined using broth microdilution based on CLSI M07-A9 protocol [29]. First, the dressings were immersed in MH broth with a concentration of 12 cm^2^/mL and incubated for 24 h. The total silver content of PCLGelAg membrane was measured by atomic absorption spectrophotometry. Then, the extracted solutions were diluted in series of 10^−1^ and tested against *P. aeruginosa* ATCC 9027 and *S. aureus* ATCC 25,923 with a density of approximately 10^7^ CFU/well. After 24 h of incubation at 37 °C, the MIC values were recorded. For the MBC evaluation, 2 µL of the tested solution from wells that had antibacterial agent concentration larger than or equal to MIC values was added onto a new MH agar plate and incubated for another 24 h. The MBC values were defined when there was no microbial growth observed.

#### 2.2.3. Time-Kill Assay

Time-kill assay was performed as recommended in Japanese Industrial Standard (JIS L 1902:2002) [30] to assess antimicrobial efficacy, maximal killing rate and speed of kill of four samples: PCLGelAg, Aquacel, UrgoTul, and sterile plasma-treated PCL as a control. The overnight suspensions of *P. aeruginosa* ATCC 9027 and *S. aureus* ATCC 25,923 were measured at 600 nm via spectroscopy and their turbidity was adjusted to 0.5 McFarland standard. Then, each piece of the dressings (1.75 cm× 1.75 cm) was loaded with 100 µL of the bacterial solution and incubated at 37 °C. After a specific time point (1, 4, 12, and 24 h), samples were taken out and vortexed in 5 mL ice-cold saline solution supplemented with 0.2% TWEEN 20 to take the surviving bacteria. The saline solution was serial diluted and the number of survived organisms was obtained by using a standard plate count procedure on MH agar plate. The log reduction was calculated as follow:

Log reduction = Log_10(control)_ – Log_10(sample)_

### 2.3. In Vivo Study

#### 2.3.1. Burn Device

**Figure 1:**
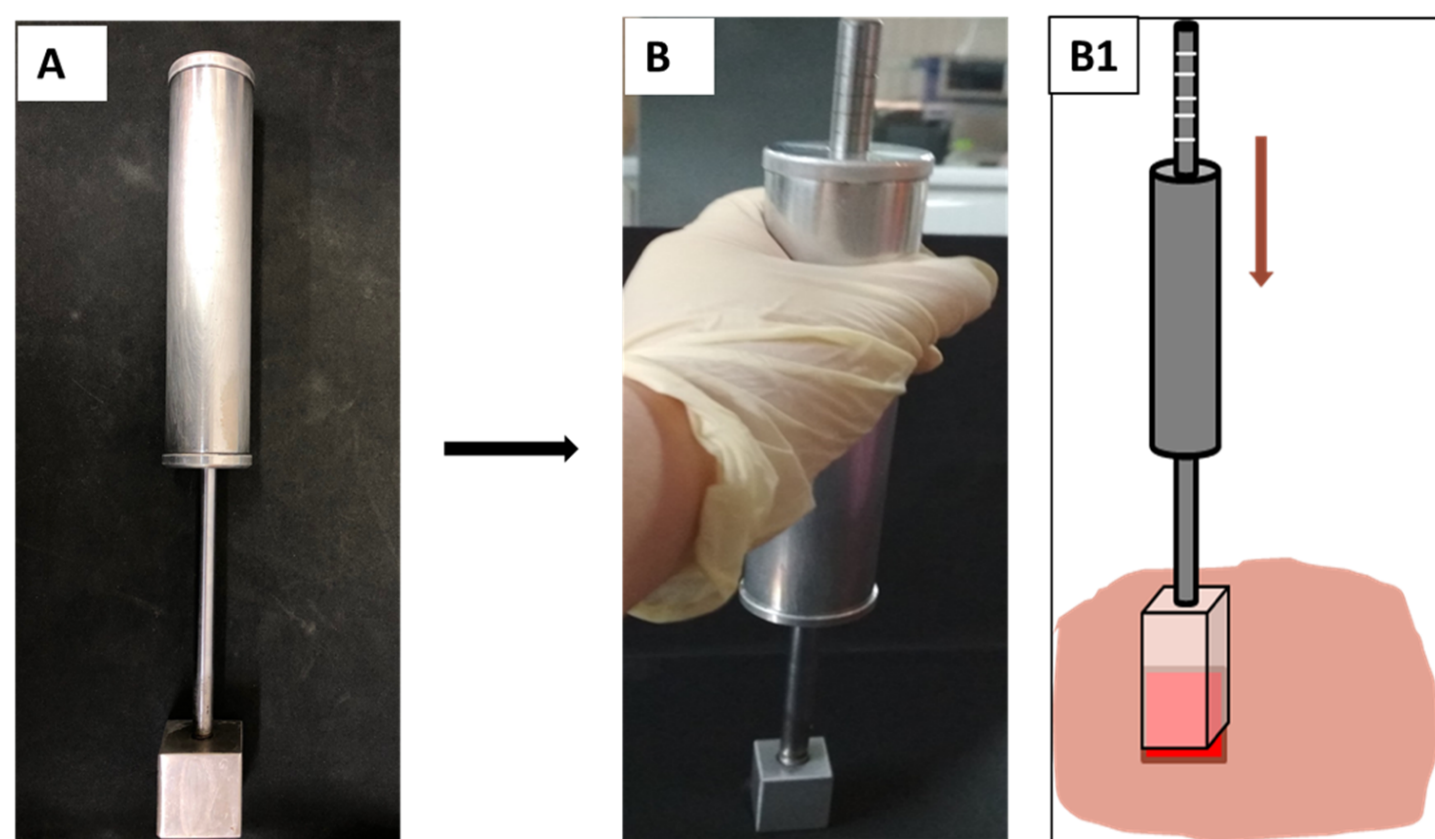
Pressure-controlled burn device (**A**) at normal condition and (**B**) when it is used to create burn wounds. (**B1**) The handle moves downward and the grade lines on the stick indicate the applied force.

A special burn device was designed to control the pressure applied to the pig skin. The target compression force was 10 N in punch. The device included an aluminum block with a 4 cm^2^ contacting surface attached to a long handle (Figure 1A). Inside the handle was a spring attached to the long stick that created elastic force when being compressed. On the stick, there were graded lines to indicate the applied pressure. When applying the device to the pig skin, the pressure made the spring compress and as the handle moved downward along the axis. As the handle went down, the gradelines on the stick appealed and the created force could be determined and controlled exactly throughout the whole experiment (Figure 1(B,B1)).

#### 2.3.2. Creation of Burn Wounds in A Porcine Model

**Figure 2:**
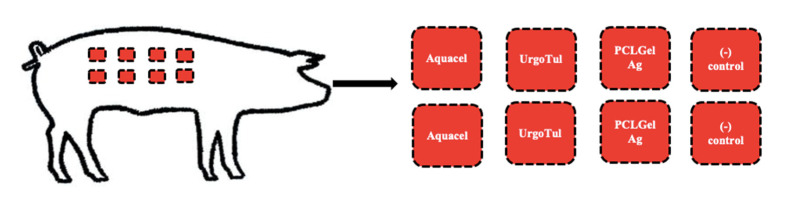
The schematic of burn wound treatment in the porcine model.

The process of creating burn wounds followed the protocol of Adam J. Singer and Steve A. McClain in the book titled Wound Healing Methods and Protocols [10]. Three male pigs weighing 20–25 kg were used in this experiment. At the beginning, 2 mg/kg of xylazine was administered intravenously as the sedative drug and 5 mg/kg of ketamine was injected after 20 min. Isoflurane gas was also used to assist the anesthesia process. For each animal subject, eight burn wounds were created at the location of each marked square (Figure 2) using the burn device. The device was preheated in boiling water for 4 min then pressed on the pig’s skin surface at 10 N and held in place for 20 s. These wounds were dressed with normal sterile gauze as a negative control, with Aquacel and UrgoTul dressings as positive controls, and with PCLGelAg membranes. New dressings were replaced once every 24 h. All the animal works in this study were approved by Animal Ethics Committees in Nong Lam University (Approval No. 20190908NLU) and performed by qualified veterinaries at a Veterinary hospital—Nong Lam University.

#### 2.3.3. Wound Closure Observation

The burn wound areas were recorded on day 0, 5, 10, and 20 post-implantation. The wound size was measured and the wound closure rate was calculated by using the following equation:

$$\%\;\mathrm{wound}\;\mathrm{closure}\;\mathrm{rate}\;=\frac{A_{t1}-A_{t2}}{A_{t1}}\times 100\%$$

where A_t1_ is the wound size on the 5th day and A_t2_ is the wound size on the 20th day.

#### 2.3.4. Histological Analysis

After 20 days, the pigs were sacrificed. Harvested skin samples were fixed in 10% *v/v* formaldehyde solution, followed with xylene and an increasing series of ethanol (30%, 50%, 60%, 70%, 80%, 90%, and 100%), then finally embedded in paraffin. Formaldehyde, xylene, ethanol and paraffin were purchased from Xilong Chemical Co., Ltd. (Shantou, China). To observe the histology of the wounds, samples were cut and stained with Hematoxylin and Eosin (H&E) and Masson’s Trichrome (MT) to evaluate skin regeneration of each dressing.

### 2.4. Statistical Analysis

All experiments were done in triplicate for in vitro study and 6 replicates for in vivo study (on 3 porcine models), unless stated otherwise. Data were demonstrated as mean ± SD (standard deviation) and statistical comparison was calculated by using SPSS Statistics software (IBM Corp, NY, USA). One-way ANOVA followed by independent *t*-tests were used to compare differences between samples. The differences were regarded as significant if the *p*-value was <0.05.

## 3. Results

### 3.1. Agar Diffusion

**Figure 3:**
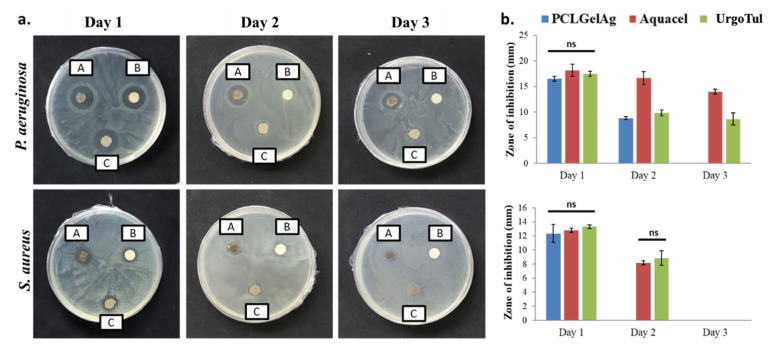
(**a**) Inhibition zones and (**b**) average zone diameters of the three wound dressing disks: (A) Aquacel, (B) PCLGelAg, and (C) UrgoTul against *P. aeruginosa* and *S. aureus.* The disks were transferred to a new agar plate every 24 h for 3 days. ns: not significant.

**Figure 4:**
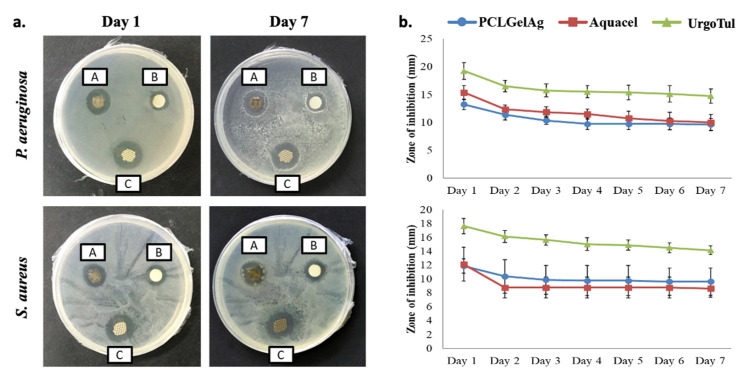
(**a**) Inhibition zones and (**b**) average zone diameters of the three wound dressing disks: (A) Aquacel, (B) PCLGelAg, and (C) UrgoTul against *P. aeruginosa* and *S. aureus*. The disks were observed every 24 h for 7 days.

Figure 3 shows the antibacterial activity of the disks when being moved to a new inoculated plate every 24 h in 3 consecutive days. In general, all of the dressings were able to inhibit both strains of bacteria with no significant difference in terms of zone diameter after 24 h of incubation. On the next day, the effect of Aquacel on *P. aeruginosa* was nearly the same (*p* > 0.05), while for the other dressings, the zones were reduced by half. On day 3, only Aquacel and UrgoTul remained active. It was further noted that the inhibition zones against *S. aureus* were significantly smaller than *P. aeruginosa.* Moreover, *S. aureus* was not inhibited by any dressings on the final day of the test. Figure 4 demonstrates the average inhibition zones that were observed every 24 h in 7 days of the three dressings. It can be observed from the graph that for both bacteria strains, there was a significant reduction in inhibition zones between day 1 and day 2. However, the zones reduced gradually (*p* > 0.05) in the following days.

### 3.2. Broth Microdilution Method

**Figure 5:**
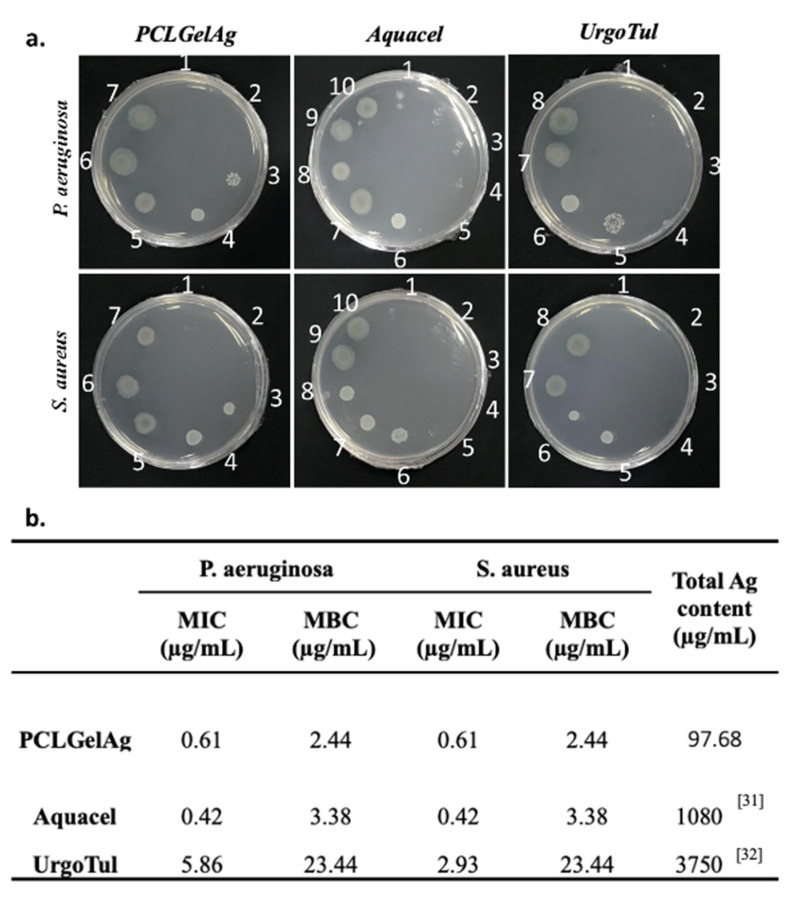
Antibacterial activity of the PCLGelAg, Aquacel and UrgoTul dressings against *P. aeruginosa* and *S. aureus* determined by the broth microdilution method. (**a**) Wells that showed no visible bacterial growth will be sampled and cultured on a new MH agar plate. The numbers (from 1 to 10) were the values of x in 2^−x^ dilution. (**b**) Minimum inhibitory concentration (MIC), minimum bactericidal concentration (MBC) values and the total silver content of PCLGelAg, Aquacel [31], and UrgoTul [32] dressings. The values represent silver concentrations.

The MIC and MBC values of the PCLGelAg, Aquacel and UrgoTul dressings against two strains of bacteria were determined through the broth microdilution method (Figure 5). When comparing the MIC values of the dressings, it can be noticed that Aquacel was the most effective in inhibiting the growth of both tested bacteria species (0.42 µg/mL), followed up by PCLGelAg (0.61 µg/mL). Being the least effective against both bacterial strains with the highest MIC, UrgoTul’s antibacterial effect against *P. aeruginosa* (5.86 µg/mL) was lower compared to *S. aureus* (2.93 µg/mL). On the other hand, PCLGelAg was proven to be the most active agent with the lowest MBC value against both *P. aeruginosa* and *S. aureus*, which was 2.44 µg/mL for both strains. By contrast, UrgoTul was the least effective with an MBC of 23.44 µg/mL. 

### 3.3. Time-Kill Assay

**Figure 6:**
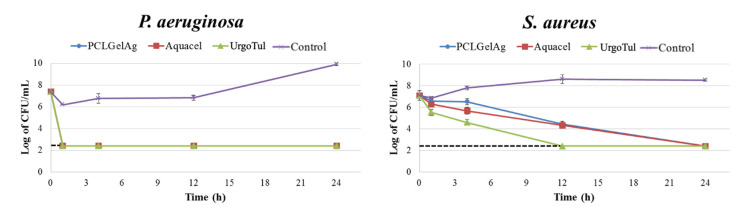
Time-kill curves of PCLGelAg, Aquacel, UrgoTul, and control dressings against *P. aeruginosa* and *S. aureus*. The black dashed lines indicate the limit of detection.

Figure 6 illustrates the mean log number of bacteria recovered from the dressings after 1 h, 4 h, 12 h, and 24 h of treatment. According to JIS L 1902:2002, a log reduction < 0.5 is rated as no antibacterial activity, 0.5 ≤ log reduction ≤ 1 as slight, 1 < log reduction ≤ 3 as significant, and a log reduction > 3 as a strong antibacterial activity. The assay confirmed the bactericidal effect (log reduction > 3.8) of all three dressings against *P. aeruginosa* within the first hour of testing and the activity lasted for at least 24 h. Against the *S. aureus* strain, after 1 h, UrgoTul dressings showed significant antibacterial effect (1.33) while Aquacel showed a slight outcome (0.56); however, PCLGelAg failed in preventing bacterial growth with a mean value of 0.26. After 4 h, only UrgoTul was able to kill 99.9% of the bacteria (3.2), whereas the other dressings reduced them to a significant level: PCLGelAg to 1.27 and Aquacel to 2.12. Nevertheless, from 12 h to 24 h, the dressings displayed a 99.99% killing effect against both bacteria species.

### 3.4. Wound Closure Observation

**Figure 7:**
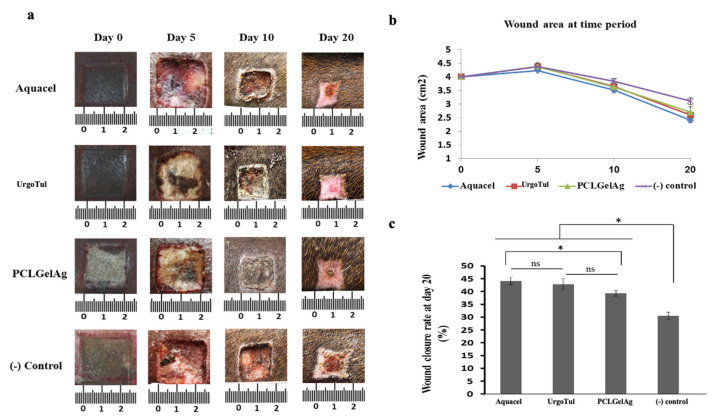
(**a**) Photographs of burn wounds treated with normal gauze (control), Aquacel, UrgoTul and PCLGelAg membranes at day 0, 5, 10, and 20; (**b**) The wound areas were measured in cm^2^ from day 0 to day 20 and presented in the line chart; (**c**) The bar chart indicates the wound closure rate after 20 days of treatment. *: *p* value < 0.05. ns: not significant.

Figure 7 shows the macroscopic observation of burn wounds and displays the healing rate among these dressings in a period of 20 days. A few seconds after the deep partial-thickness burns were created, the epidermis was damaged severely. Under the epidermis, the wounds appeared with a pale white color. The initial area of all wounds was 4 cm^2^. However, on the 5th day, there was an extension in the wound area in all four treatments. In detail, wounds treated with UrgoTul and Aquacel extended to about 4.2 cm^2^ while those treated with PCLGelAg and normal gauze extended to 4.4 cm^2^. On the 5th day, the epidermis layer was totally removed from the wound site and wounds turned red. On the 10th day, a thick scab developed and covered the wound surface, under which a wound healing process was occurring. After 20 days, the closure rate was significantly higher in wounds treated with Aquacel and UrgoTul with the rate up to over 40%. In addition, wounds treated with PCLGelAg presented a healing rate of 38% while wounds dressed with normal gauze were only at 30%.

### 3.5. Histology Analysis

**Figure 8:**
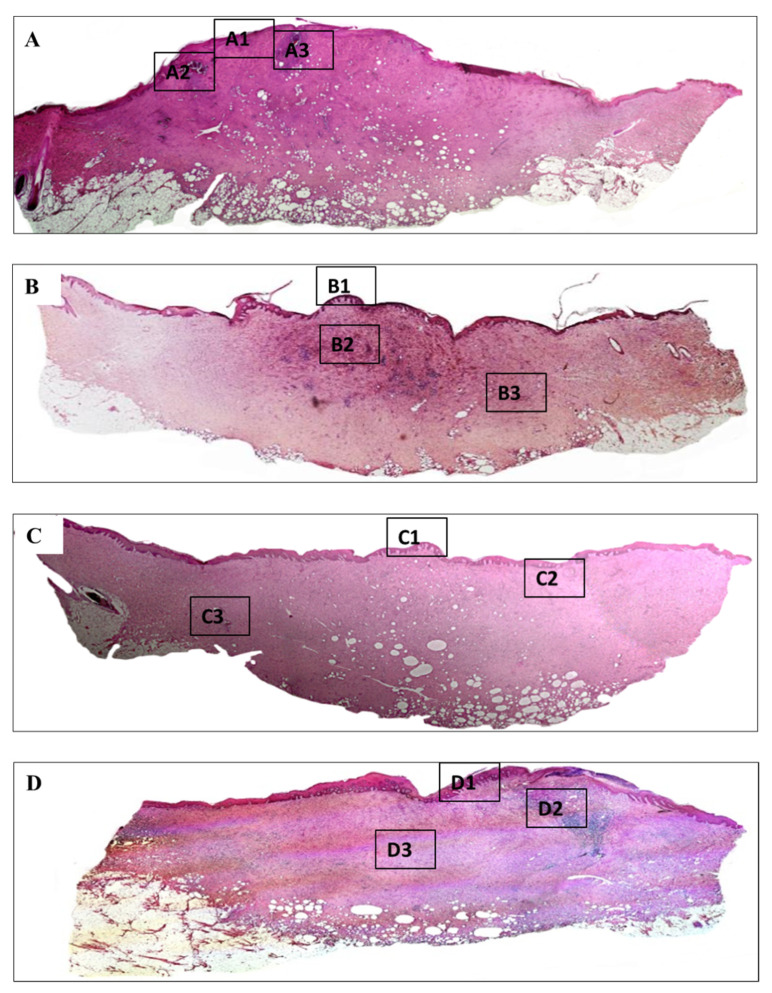
H&E stained sections of wounds treated with (**A**) normal gauze, (**B**) Aquacel, (**C**) UrgoTul, and (**D**) PCLGelAg membranes. The tissue sections are magnified at (**A1**–**D1**) the new epidermis, (**A2**,**A3**,**B2**,**B3**,**C2**,**C3**,**D2**,**D3**) inflammatory areas and some newly generated structures.

**Figure 9:**
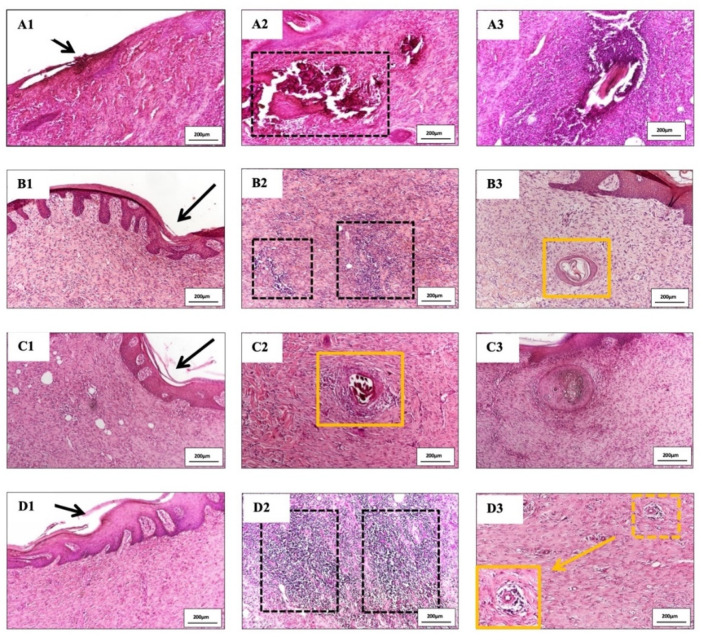
Magnified images of tissue sections of wounds treated with (**A1**–**A3**) normal gauze, (**B1**–**B3**) Aquacel, (**C1**–**C3**) UrgoTul, and (**D1**–**D3**) PCLGelAg membranes as mentioned above. The black arrows in figures (**A1**–**D1**) show new formed epidermis. The black dot border squares indicate the inflammatory areas (**B2**,**D2**) and necrosis area (**A2**) of the wounds. Other images show new regenerated under skin structures including hair follicles, new blood vessels (yellow square boxes).

**Figure 10:**
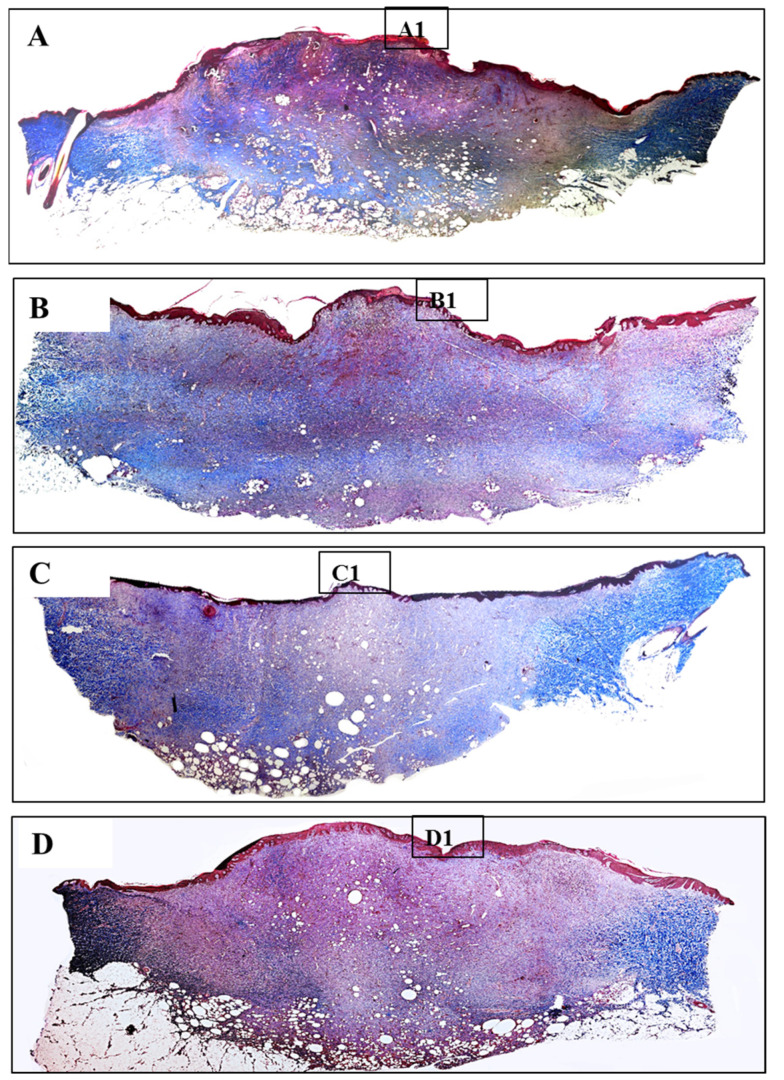
MT-stained sections of wounds treated with (**A**) normal gauze, (**B**) Aquacel, (**C**) UrgoTul, and (**D**) PCLGelAg membranes. The black square boxes (**A1**–**D1**) show the epidermis and a part of the dermis in the center of the wounds, respectively.

Meanwhile, histopathology of burn injury is characterized by three main features, including the deposition of collagen, loss of under skin structure such as hair follicle, and vascular of secretion glands, which can be observed clearly by H&E (Figure 8) and MT staining (Figure 10). The depth of all the wounds reached the subcutaneous layer and nearly became full-thickness burn injury. Wounds treated with normal gauzes showed incomplete regeneration of epidermis, under which there were many infiltrated neutrophils (Figure 9(A1)). The dermis increased in thickness with the high proliferation of fibroblasts and a high density of inflammatory cells that infiltrated in a large area. In addition, at the dermis layer, signs of necrosis areas (Figure 9(A2)) and newly formed hair follicles (Figure 9(A3)) were observed. Surroundings of the hair follicles were filled with lymphocytes and fibroblasts.

On the other hand, wounds treated with Aquacel and UrgoTul resulted in better recovery. The epidermis was completely regenerated (Figure 9(B1,C1)) and there was no sign of infection or necrosis inside. For Aquacel samples, there was a high proliferation of fibroblasts. In the middle of the tissue, a small area still displayed the inflammatory cells, including lymphocytes and neutrophils (Figure 9(B2)). For UrgoTul, the wound recovered even better with a new completely-regenerated epidermis layer (Figure 9(C1)) and very few inflammatory cells were found inside the tissue (Figure 9(C2)). A new hair follicle was completely formed in Figure 9(C3). In wound treated with PCLGelAg membranes, the epidermis appeared totally restructured (Figure 9(D1)). On the right side of the wound, there was a small area where a highly dense group of inflammatory cells (neutrophils and plasmocytes) infiltrated into. No sign of infection or necrosis was observed (Figure 9(D2)). Moreover, wounds treated with Aquacel, UrgoTul, and PCLGelAg showed signs of new blood vessel formation (Figure 9(C2,B3,D3)). 

**Figure 11:**
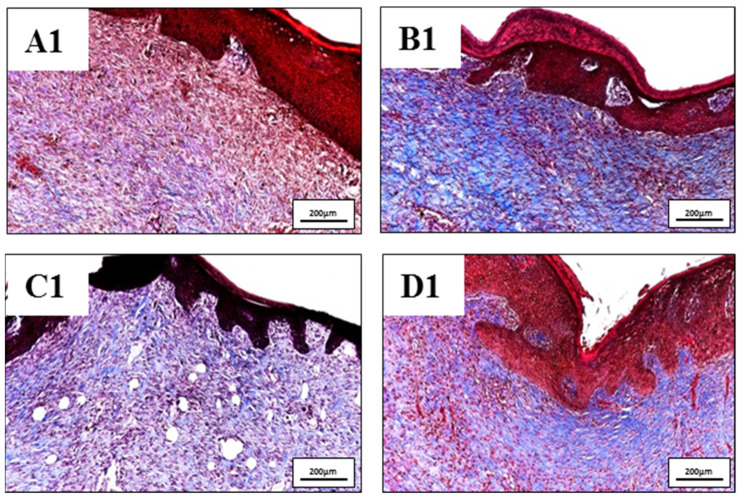
Magnified images of tissue sections of wounds treated with (**A1**) normal gauze, (**B1**) Aquacel, (**C1**) UrgoTul, and (**D1**) PCLGelAg membranes as mentioned above in Figure 10. The images compared the regeneration of new collagen structure in the dermis layer of different wounds.

In nature, skin tissue has three layers which are epidermis, dermis and subcutaneous. The collagen structure inside the tissue presents in the form of fibrous bundles with large diameters and dense fibers which were well-organized and aligned in each bundle [33,34]. Burn leads to the destruction of the whole collagen structure under the skin, so that the regeneration of new collagen also helps to evaluate the effectiveness of the healing process. In burn injuries, the skin lost its original collagen structure, collagen fibers were aggregated together and formed an amorphous structure which can be clearly observed in the center of all wounds (Figure 10). Collagen structure primarily regenerated at the wound edges and slowly moved toward inside. Wounds treated with silver-containing commercial products (Aquacel and UrgoTul) resulted in a higher rate of collagen formation which is presented with a more intense blue color inside the wounds (Figure 11(B1,C1)). The new collagen regenerated throughout all of the wounds showed that these wounds were steadily healing. In contrast, collagen density was found lower in wounds treated with PCLGelAg (Figure 11(D1)) and normal gauze (Figure 11(A1)), especially in the center of the wounds.

## 4. Discussion

Generally, an open wound is susceptible to be colonized by the pathogens available in the surrounding environment. Therefore, once infected, it is critical to have an antimicrobial dressing acted as a barrier to prevent more bacterial penetration, minimize spreading infection and facilitate wound healing [35]. In this study, the longevity of the dressings’ decontamination ability was evaluated through two disk diffusion models as shown in Figure 3 and Figure 4: the first one is to find out whether the dressings still inhibit bacteria if they are moved to another inoculated agar plate daily, whereas the latter is to see if the inhibition zones are still present after a week on one agar plate without any transfer. Even though the agar diffusion test tightly depends on the diffusion characteristics of the antimicrobial agent, silver might interact with the agar medium, leading to the non-linear relationship between silver release and diffusion [36]. This is probably why the difference in inhibition zone sizes of the dressings after 24 h of incubation was not significant despite having differences in silver form and concentration (Figure 3). On the other hand, the reduction in zone diameter after each transfer (Figure 4) is in agreement with a study of Kostenko et al., where they have proven that silver dressings with hydrophilic based materials showed short-term efficacy in a plate-to-plate transfer assay [37]. The results from both Figure 3 and Figure 4 also revealed that *P. aeruginosa* was more sensitive with silver than *S. aureus* as the inhibition zones of samples against *P. aeruginosa* were larger and the antibacterial efficacy was exhibited longer. In specific, Gram-positive bacteria like *S. aureus* have a thicker cell wall and a higher amount of negatively charged peptidoglycan, which prevents the silver ions from entering deeper into the inner side of the cell membrane, thus limiting their action and giving the bacteria an opportunity to develop silver resistance [38]. Furthermore, the antibacterial effect of silver is concentration-dependent and time-dependent. The silver content of PCLGelAg was the least among the three dressings (8.14 µg/cm^2^); therefore, it was not enough to maintain the inhibition zone for more than one day against *S. aureus* and two days against *P. aeruginosa,* though no bacterial growth was found in the area under the disc. For the second agar diffusion model, longer use without dressing change resulted in the regrowth of bacteria, though only the zone reduction on day 2 was significant (Figure 4). This failure in bacterial suppression might be due to the low level of dressing hydration and the insufficient amount of silver released. 

Since the zone of inhibition is not proportional to the release of silver, the agar diffusion test alone is not enough to reflect the antimicrobial efficacy of the dressings. Hence, the broth microdilution method was employed to determine their MIC and MBC values. The result from Figure 5, despite not correlating with the zone sizes in the agar diffusion test, indicated that PCLGelAg demonstrated a good antibacterial activity with low MIC (0.61 µg/mL) and MBC (2.44 µg/mL) when being tested against both bacterial strains. The value is similar to previous studies [39,40], where a silver concentration of as little as 1–2 µg/mL was adequate to inhibit bacteria growth or achieve bactericidal action. The in vitro effectiveness of PCLGelAg against bacteria was comparable to Aquacel but significantly better than UrgoTul.

In order to obtain a closer look at the killing kinetics of the dressings, the time-kill assay was carried out. Figure 6 again reinforced the fact that *P. aeruginosa* has a higher susceptibility to silver than *S. aureus* in this study, as the number of its colonies fell under the detection limit just one hour after getting into contact with the dressings. Aside from the cell wall thickness and composition diversities, water deprivation, to which Gram-negative bacteria is much more vulnerable, also plays a role in this difference in antibacterial efficacy when compared to Gram-positive species [41]. It should be noted that the composition, especially the silver form, of each dressing is different: UrgoTul contains carboxymethyl cellulose (CMC), petroleum jelly and silver sulfadiazine fused together in a polyester mesh [32]; Aquacel is composed of CMC-based fibers impregnated in ionic silver [31]; while PCLGelAg is an electrospun PCL membrane coated with gelatin/silver nanoparticles. However, both CMC and plasma-treated PCL possess high fluid absorption capacity. As a result, when the dressings absorb fluid and swell, bacteria available in the MH medium might be trapped inside their polymer matrix, leading to the reduction in bacteria colonies remained after being collected between 0 h and 1 h. On the other hand, even though PCLGelAg expressed a slower speed of kill against *S. aureus*, the dressing was able to reach maximal killing rate after 12 h of bacteria exposure and sustain the effect for at least 24 h. Indeed, the antimicrobial activity is further affected by the diverse silver species [42], which are in charge of the interaction with bacteria, and base materials, which manage the silver released duration and concentration [37]. Combining with the data from the agar diffusion assay and microdilution method, it is suggested that PCLGelAg should be used topically and changed daily to maintain the highest bactericidal performance.

In the in vivo study, a normal deep burn wound has three zones of damage including a zone of hyperemia, a zone of stasis and a zone of coagulation from the outermost to the center respectively based on the severity of tissue destruction and alteration in blood flow [7]. Among these zones, the zone of coagulation is caused when the skin is destroyed by coming into contact with a hot thermal source. Tissues in the coagulation zone are the most suffered with a high rate of necrosis and extensive protein denaturation, and take a longer time to heal. Tissue destruction and necrosis not only occur in the coagulation zone. After a burn injury happens, within 24–48 h, the burn area could be wider and deeper, which affects the stasis zone outside the coagulation zone. This helps explain the increase in wound area found in all four PCLGelAg, Aquacel, UrgoTul, and control samples in the first 5 days (Figure 7). The delay-necrosis tissue is not well understood, but hypoxia and ischemia that lead to lack of intervention are believed to be main reasons for this phenomenon [2,7,43]. The outermost zone is the zone where hyperemia occurs, which is characterized by vasodilation and inflammation but low-risk necrosis. This zone is also where wound healing begins [3,43].

The healing process of a burn wound occurs in four overlapping phases, beginning with the homeostasis phase right after the tissue is damaged [44]. This phase is characterized by platelet aggregation, blood clotting, and vascular restriction. After that, the immune system then responses to the signal of the body and the inflammatory phase occurs. At the beginning, the inflammatory cells, including neutrophils and monocytes (which can differentiate into macrophage), migrate to the injury site to protect the body from harmful pathogens, prevent local infection and help to degrade necrotic tissue. During the inflammatory phase, the proliferation phase co-occurs and overlaps the inflammatory phase. This phase includes three main steps which are re-epithelialization, angiogenesis, and formation of granulation tissue. In this phase, keratinocytes and fibroblasts migrate over the wound to assist the healing process as well as produce ECM to regenerate skin structure and revascularization. The final phase is the remodeling phase, where the burn wound completely heals and forms scars as collagen and elastin continue to deposit and reform. This phase lasts for several months or years [2,44,45]. According to the results above, wounds treated with UrgoTul and Aquacel showed better recovery, which was indicated through higher wound closure and collagen formation rates, new skin structure formation and a low density of inflammatory cells inside the wounds. Although inflammation plays an important role in the healing process, prolonged inflammation could cause impaired wound healing that delays tissue repair [2,45,46]. On the other hand, although the healing rate was not as high as those treated with UrgoTul and Aquacel, PCLGelAg samples still showed better recovery of damaged skin when compared with normal gauze treatment and effectiveness in antibacterial properties. The effectiveness in wounds treated with silver-containing dressings is believed to be due to the high antibacterial properties of silver that were extensively proved in previous studies. Moreover, besides the broad-spectrum antibacterial activity, silver also helps to modulate inflammatory activity by reducing cytokine release together with creating a decrease in lymphocyte and mast cell infiltration to the wound bed. In detail, silver suppresses the production of many cytokines such as TNF-α, IL-6, and TGF-β that help to reduce inflammatory cell accumulation and increase the level of IL-10, VEGF, and IFN-λ, thereby promote the wound healing process [12,45,46,47,48]. This explains the better wound healing performance and reduced inflammation on the pig model of three silver-containing samples over the control.

It could be seen here and from our previous study [28] that the supportive effect of gelatin coating layer on proliferation of cells was overshadowed by the effect of AgNPs. This explains why PCLGelAg membrane in general did not result in better recovery compared to the other two commercial products. However, when compared to negative control, PCLGelAg membrane still significantly enhanced wound healing fashion. This was due to multiple effects of AgNPs on would healing biology. First of all, beside antibacterial effects, AgNPs were proven to markedly induce KGF-2 expression which suppressed the phosphorylation activation of p38 MAPK and led to a reduction of IL-6 produced by keratinocytes, and subsequently less neutrophil infiltration in the burn wounds [45]. This was also confirmed in our data as the negative control wounds were flooded with neutrophils and plasmacytes in the absence of AgNPs even 20 days post-injury, which was an indication of the prolonged inflammation phenomena. Secondly, AgNPs were also proven to drive the differentiation of fibroblasts to myofibroblasts [49], which again confirmed by the faster wound contracting rate of three silver-containing samples. Another important point was the effect of AgNPs in supporting the synthesis of collagen during wound healing. Till now, the mechanism of how AgNPs affect collagen synthesis is related to the TGFβ1-Smad2/3 signaling pathway. This was clearly proven in the in vivo study of Chen et al. [50], where dressings with AgNPs yielded a significant increase in the synthesis of type I collagen compared to normal gauge without AgNPs and the introduction of TGFβ1 inhibitor did reduce a significant amount of newly formed collagen. Moreover, Sushovan et al. showed that single AgNPs treated wounds did not show increasement while daily applied AgNPs significantly enhanced collagen deposition on the incision site [51]. Comparing to our Masson’s Trichrome staining data, the practice of applying dressings daily onto wound sites did result in a significant increase in collagen formation for three silver-containing dressings compared to the negative normal gauge controls. In general, it is clear that the PCLGelAg membrane which displayed antibacterial and cytocompatible properties in our previous study, has now further been highlighted to possess advantageous effects in treating severe burn injuries and is a promising candidate for clinical applications.

## 5. Conclusions

Comparing to two other commercial silver-containing wound dressing products (Aquacel and UrgoTul), the multi-coated PCLGelAg membrane was proved to possess long-lasting antibacterial efficiency and wound healing properties for burn injury. Agar diffusion assays showed that PCLGelAg remained its efficiency in inhibiting Gram-negative and Gram-positive bacteria in 24 h and prolonged the inhibitory effect for 7 days. The PCLGelAg membrane also showed the highest effectiveness in killing bacteria with the lowest MBC value at 2.44 µg/mL against both strains, while the Aquacel membrane possessed the lowest MIC value at 0.42 µg/mL. In addition, all of the silver-containing dressings demonstrated highly effective antibacterial ability, which killed 99.99% of *P. aeruginosa* in the first hour and *S. aureus* within 12 h after application, and the effect could last for 24 h. In vivo experiment on a pig model demonstrated all three PCLGelAg, Aquacel and UrgoTul dressings supported wound healing as well as prevented infection and inflammation when compared to the control group, indicating practical applications of the PCLGelAg membrane in the treatment of burn injuries.

## Data Availability

All the data will be available to the readers.

## References

[1] Alemayehu S., Afera B., Kidanu K., Belete T. (2020). Management Outcome of Burn Injury and Associated Factors among Hospitalized Children at Ayder Referral Hospital, Tigray, Ethiopia. Int. J. Pediatr..

[2] Rowan M.P., Cancio L.C., Elster E.A., Burmeister D.M., Rose L.F., Natesan S., Chan R.K., Christy R.J., Chung K.K. (2015). Burn wound healing and treatment: Review and advancements. Crit. Care.

[3] Church D., Elsayed S., Reid O., Winston B., Lindsay R. (2006). Burn Wound Infections. Clin. Microbiol. Rev..

[4] Lawrence J.C., Bull J.P. (1976). Thermal Conditions which Cause Skin Burns. Eng. Med..

[5] Nielson C.B., Duethman N.C., Howard J.M., Moncure M., Wood J.G. (2017). Burns: Pathophysiology of Systemic Complications and Current Management. J. Burn Care Res..

[6] Wang Y., Beekman J., Hew J., Jackson S., Issler-Fisher A.C., Parungao R., Lajevardi S.S., Li Z., Maitz P.K. (2018). Burn injury: Challenges and advances in burn wound healing, infection, pain and scarring. Adv. Drug Deliv. Rev..

[7] Evers L.H., Bhavsar D., Mailänder P. (2010). The biology of burn injury. Exp. Dermatol..

[8] Jeschke M.G., Van Baar M.E., Choudhry M.A., Chung K.K., Gibran N.S., Logsetty S. (2020). Burn injury. Nat. Rev. Dis. Primers.

[9] Shakespeare P. (2001). Burn wound healing and skin substitutes. Burns.

[10] Spanholtz T.A., Theodorou P., Amini P., Spilker G. (2009). Severe burn injuries: Acute and long-term treatment. Dtsch. Ärzteblatt Int..

[11] Dai T., Huang Y., Sharma S.K., Hashmi J.T., Kurup D.B., Hamblin M.R. (2010). Topical antimicrobials for burn wound infections. Recent Pat. Anti-Infect. Drug Discov..

[12] Gunasekaran T., Nigusse T., Dhanaraju M.D. (2011). Silver Nanoparticles as Real Topical Bullets for Wound Healing. J. Am. Coll. Clin. Wound Spec..

[13] Zhong W. (2015). Efficacy and toxicity of antibacterial agents used in wound dressings. Cutan. Ocul. Toxicol..

[14] Filipović U., Dahmane R.G., Ghannouchi S., Zore A., Bohinc K. (2020). Bacterial adhesion on orthopedic implants. Adv. Colloid Interface Sci..

[15] Murphy P.S., Evans G.R.D. (2012). Advances in Wound Healing: A Review of Current Wound Healing Products. Plast. Surg. Int..

[16] Carsin H., Wasserman D., Pannier M., Dumas R., Bohbot S. (2004). A silver sulphadiazine-impregnated lipidocolloid wound dressing to treat second-degree burns. J. Wound Care.

[17] Heyneman A., Hoeksema H., Vandekerckhove D., Pirayesh A., Monstrey S. (2016). The role of silver sulphadiazine in the conservative treatment of partial thickness burn wounds: A systematic review. Burns.

[18] Nowack B., Krug H.F., Height M. (2011). 120 Years of Nanosilver History: Implications for Policy Makers. Environ. Sci. Technol..

[19] Khansa I., Schoenbrunner A.R., Kraft C.T., Janis J.E. (2019). Silver in Wound Care—Friend or Foe? A Comprehensive Review. Plast. Reconstr. Surg. Glob. Open.

[20] Wasef L.G., Shaheen H.M., El-Sayed Y.S., Shalaby T., Samak D.H., El-Hack M.E.A., Al-Owaimer A., Saadeldin I., El-Mleeh A., Ba-Awadh H. (2020). Effects of Silver Nanoparticles on Burn Wound Healing in a Mouse Model. Biol. Trace Elem. Res..

[21] Galandáková A., Frankova J., Ambrozova N., Habartová K., Pivodová V., Zálešák B., Šafářová K., Smékalová M., Ulrichova J. (2016). Effects of silver nanoparticles on human dermal fibroblasts and epidermal keratinocytes. Hum. Exp. Toxicol..

[22] Nguyen T.D., Nguyen T.T., Ly K., Tran H., Nguyen T.T.N., Vo M.T., Ho H.M., Dang N.T.N., Vo V.T., Nguyen D.H. (2019). In Vivo Study of the Antibacterial Chitosan/Polyvinyl Alcohol Loaded with Silver Nanoparticle Hydrogel for Wound Healing Applications. Int. J. Polym. Sci..

[23] Ramadhan M.A.K., Balasm A.N., Kadhem S.B., Al-Saedi H.F. (2020). Effect of Silver Nanoparticles on Healing of Third-Degree Burns Infected with Pseudomonas Aeruginosa in Laboratory Mice. Maced. Vet. Rev..

[24] Pang S., Gao Y., Wang F., Wang Y., Cao M., Zhang W., Liang Y., Song M., Jiang G. (2020). Toxicity of silver nanoparticles on wound healing: A case study of zebrafish fin regeneration model. Sci. Total Environ..

[25] Iljas J., Röhl J., McGovern J., Moromizato K., Parker T., Cuttle L. (2021). A human skin equivalent burn model to study the effect of a nanocrystalline silver dressing on wound healing. Burns.

[26] Thanh N.T., Hieu M.H., Phuong N.T.M., Thuan T.D.B., Thu H.N.T., Thai V.-P., Minh T.D., Dai H.N., Vo V.T., Thi H.N. (2018). Optimization and characterization of electrospun polycaprolactone coated with gelatin-silver nanoparticles for wound healing application. Mater. Sci. Eng..

[27] Ho M.H., Do T.B.-T., Dang N.N.-T., Le A.N.-M., Ta H.T.-K., Van Vo T., Nguyen H.T. (2019). Effects of an Acetic Acid and Acetone Mixture on the Characteristics and Scaffold–Cell Interaction of Electrospun Polycaprolactone Membranes. Appl. Sci..

[28] Nguyen T.N., Do T.B., Ho M.H., Tran N.M., Dang N.N., Do T.M., Nguyen H.T., Phan T.B., Tran Q.N., Van Vo T. (2021). Investigating the effect of multi-coated hydrogel layer on characteristics of electrospun PCL membrane coated with gelatin/silver nanoparticles for wound dressing application. J. Biomed. Mater. Res. Part A.

[29] Clinical and Laboratory Standards Institute (2012). Methods for Dilution Antimicrobial Susceptibility Tests for Bacteria that Grow Aerobically. Approved Standard.

[30] The Japanese Industrial Standards (2002). Testing for Antibacterial Activity and Efficacy on Textile Products.

[31] Parsons D., Bowler P.G., Myles V., Jones S. (2005). Silver antimicrobial dressings in wound management: A comparison of antibacterial, physical, and chemical characteristics. Wounds.

[32] White R., Cowan T., Glover D. (2015). Supporting Evidence-Based Practice: A Clinical Review of TLC Healing Matrix.

[33] Summerfield A., Meurens F., Ricklin M.E. (2015). The immunology of the porcine skin and its value as a model for human skin. Mol. Immunol..

[34] Debeer S., Le Luduec J.-B., Kaiserlian D., Laurent P., Nicolas J.F., Dubois B., Kanitakis J. (2013). Comparative histology and immunohistochemistry of porcine versus human skin. Eur. J. Dermatol..

[35] Simões D., Miguel S.A.P., Ribeiro M., Coutinho P., Mendonça A., Correia I.J. (2018). Recent advances on antimicrobial wound dressing: A review. Eur. J. Pharm. Biopharm..

[36] Vaughan J., Benson R., Vaughan K., Farrar D. (2011). Assessing the effectiveness of antimicrobial wound dressings In Vitro. Advanced Wound Repair Therapies.

[37] Kostenko V., Lyczak J., Turner K., Martinuzzi R.J. (2010). Impact of silver-containing wound dressings on bacterial biofilm viability and susceptibility to antibiotics during prolonged treatment. Antimicrob. Agents Chemother..

[38] Dakal T.C., Kumar A., Majumdar R.S., Yadav V. (2016). Mechanistic basis of antimicrobial actions of silver nanoparticles. Front. Microbiol..

[39] Wilkinson L.J., White R.J., Chipman J.K. (2011). Silver and nanoparticles of silver in wound dressings: A review of efficacy and safety. J. Wound Care.

[40] Yuan Y.-G., Peng Q.-L., Gurunathan S. (2017). Effects of silver nanoparticles on multiple drug-resistant strains of staphylococcus aureus and pseudomonas aeruginosa from mastitis-infected goats: An alternative approach for antimicrobial therapy. Int. J. Mol. Sci..

[41] Wiegand C., Abel M., Ruth P., Elsner P., Hipler U.-C. (2015). In Vitro assessment of the antimicrobial activity of wound dressings: Influence of the test method selected and impact of the pH. J. Mater. Sci..

[42] Szweda P., Gorczyca G., Tylingo R. (2018). Comparison of antimicrobial activity of selected, commercially available wound dressing materials. J. Wound Care.

[43] Rose L.F., Chan R.K. (2016). The Burn Wound Microenvironment. Adv. Wound Care.

[44] Gurtner G.C., Werner S., Barrandon Y., Longaker M.T. (2008). Wound repair and regeneration. Nature.

[45] Zhang K., Lui V.C.H., Chen Y., Lok C.N., Wong K.K.Y. (2020). Delayed application of silver nanoparticles reveals the role of early inflammation in burn wound healing. Sci. Rep..

[46] You C., Li Q., Wang X., Wu P., Ho J.K., Jin R., Zhang L., Shao H., Han C. (2017). Silver nanoparticle loaded collagen/chitosan scaffolds promote wound healing via regulating fibroblast migration and macrophage activation. Sci. Rep..

[47] Merrell J.G., McLaughlin S.W., Tie L., Laurencin C.T., Chen A.F., Nair L.S. (2009). Curcumin-loaded poly(ε-caprolactone) nanofibres: Diabetic wound dressing with anti-oxidant and anti-inflammatory properties. Clin. Exp. Pharmacol. Physiol..

[48] Atiyeh B.S., Costagliola M., Hayek S.N., Dibo S.A. (2007). Effect of silver on burn wound infection control and healing: Review of the literature. Burns.

[49] Liu X., Lee P.-Y., Ho C.-M., Lui V.C.H., Chen Y., Che C.M., Tam P.K.H., Wong K.K.Y. (2010). Silver nanoparticles mediate differential responses in keratinocytes and fibroblasts during skin wound healing. ChemMedChem.

[50] Li C.W., Wang Q., Li J., Hu M., Shi S.J., Li Z.W., Wu G.L., Cui H.H., Li Y.Y., Zhang Q. (2016). Silver nanoparticles/chitosan oligosaccharide/poly(vinyl alcohol) nanofiber promotes wound healing by activating TGFβ1/Smad signaling pathway. Int. J. Nanomed..

[51] Chowdhury S., De M., Guha R., Batabyal S., Samanta I., Hazra S.K., Ghosh T.K., Konar A., Hazra S. (2014). Influence of silver nanoparticles on post-surgical wound healing following topical application. Eur. J. Nanomed..

